# Penicillin Allergy De-labeling Results in Significant Changes in Outpatient Antibiotic Prescribing Patterns

**DOI:** 10.3389/falgy.2020.586301

**Published:** 2020-12-16

**Authors:** Thomas Hills, Nicola Arroll, Eamon Duffy, Janice Capstick, Anthony Jordan, Penny Fitzharris

**Affiliations:** ^1^Department of Immunology, Auckland District Health Board, Auckland, New Zealand; ^2^Medical Research Institute of New Zealand, Wellington, New Zealand; ^3^Health Information and Technology Service, Auckland District Health Board, Auckland, New Zealand; ^4^Depatment of Infectious Diseases, Auckland District Health Board, Auckland, New Zealand

**Keywords:** penicillin, antibiotic, allergy, hypersensitivity, prescribing, electronic dispensing data, antimicrobial stewardship

## Abstract

Unverified penicillin allergies are common but most patients with a penicillin allergy label can safely use penicillin antibiotics. Penicillin allergy labels are associated with poor clinical outcomes and overuse of second-line antibiotics. There is increasing focus on penicillin allergy “de-labeling” as a tool to improve antibiotic prescribing and antimicrobial stewardship. The effect of outpatient penicillin allergy de-labeling on long-term antibiotic use is uncertain. We performed a retrospective pre- and post- study of antibiotic dispensing patterns, from an electronic dispensing data repository, in patients undergoing penicillin allergy assessment at Auckland City Hospital, New Zealand. Over a mean follow-up of 4.55 years, 215/304 (70.7%) of de-labeled patients were dispensed a penicillin antibiotic. Rates of penicillin antibiotic dispensing were 0.24 (0.18–0.30) penicillin courses per year before de-labeling and 0.80 (0.67–0.93) following de-labeling with a reduction in total antibiotic use from 2.30 (2.06–2.54) to 1.79 (1.59–1.99) antibiotic courses per year. In de-labeled patients, the proportion of antibiotic courses that were penicillin antibiotics increased from 12.81 to 39.62%. Rates of macrolide, cephalosporin, trimethoprim/co-trimoxazole, fluoroquinolone, “other” non-penicillin antibiotic use, and broad-spectrum antibiotic use were all lower following de-labeling. Further, antibiotic costs were lower following de-labeling. In this study, penicillin allergy de-labeling was associated with significant changes in antibiotic dispensing patterns.

## Introduction

Penicillin allergy labels (PALs) are common with 8–25% of individuals labeled as allergic to penicillin, depending on the patient group studied (1). PALs are intended to protect patients from receiving antibiotics that may cause direct harm due to allergic drug reactions. However, most patients with a PAL will tolerate penicillin antibiotics because these labels are frequently inaccurate. Inaccurate PALs occur because of the overdiagnosis of antibiotic allergy in the context of non-allergic childhood rashes, misclassification of non-allergic adverse reactions, and because true IgE-mediated allergy can wane with time (2–4). A PAL itself is not benign and the presence of a PAL is associated with poor outcomes including increased length of hospital stay (5), increased risk of infection with multi-resistant organisms (5–7), increased rates of *Clostridium difficile* diarrhea (7), inappropriate antibiotic prescribing and overuse of second-line antibiotics (8), more surgical site infections (9), increased costs (10), and increased mortality (6).

Clarifying a patient's penicillin allergy status has the potential to improve their access to appropriate and safe antibiotic therapy while also contributing to antimicrobial stewardship efforts in the era of increasing antimicrobial resistance (11, 12). Assessment in a specialist antibiotic allergy clinic with access to penicillin allergy skin testing and observed oral penicillin challenges (drug provocation tests) is a widely-used approach; there is extensive evidence that this is safe and that the majority of patients assessed will tolerate penicillin antibiotics (that is, they are not allergic and can be de-labeled) (3, 12–15). Antibiotic allergy assessment and de-labeling meant 59/62 (95.2%) patients were willing to use the de-labeled antibiotic (16). However, prescribers must also be willing to prescribe the relevant antibiotic and, in some settings, the persistence of PALs despite negative observed oral penicillin challenges has been identified as a significant issue (17–19). Hospital inpatients, de-labeled during an admission when penicillin antibiotics are indicated, will typically use the penicillin antibiotic immediately (12, 20). There is a relative paucity of data on patterns of longer-term antibiotic use in de-labeled patients, particularly in the outpatient setting.

Electronic dispensing data repositories are powerful potential tools for research on community antibiotic use (21). The pattern of antibiotic use before and after assessment in our clinic is unknown. The great majority of antibiotic use occurs in the community. In New Zealand, at 95% of total antibiotic consumption, the proportion of total antibiotic consumption that occurs in the community is higher than in other countries (22). Therefore, community antibiotic consumption is a key metric in studies seeking to measure changes in antibiotic use, particularly in New Zealand.

Electronic pharmacy dispensing records allow the direct comparison of community antibiotic dispensing before and after penicillin allergy evaluation. Here, we contrast antibiotic prescribing before and after penicillin allergy de-labeling through the analysis of several years of pharmacy dispensing records, thus providing information on antibiotic use over longer periods than previously reported. We characterize the use of specific non-penicillin antibiotics that are important targets for antimicrobial stewardship efforts. Further, we compare both the antimicrobial spectrum and cost of antibiotics dispensed before and after de-labeling.

## Materials and Methods

### Inclusion and Exclusion Criteria

Adult patients who underwent an observed penicillin challenge through the Penicillin Allergy Clinic in the Department of Clinical Immunology at Auckland City Hospital between 01/01/2012 and 31/12/2017 were identified from electronic records of Immunology Day Ward attendances. There are approximately 70 penicillin challenges performed through the Antibiotic Allergy Clinic at Auckland City Hospital annually. Further, those with positive penicillin skin testing were identified from electronic records of penicillin skin testing results in the Penicillin Allergy Clinic in the same time period. Patients were excluded from the final analysis if they were aged <15 years at the time of assessment, if they had no medication entries in the TestSafe database prior to assessment, and if they had no antibiotic dispensing entries in the TestSafe database across the pre- and post-assessment periods.

### Skin Testing and Penicillin Challenge Methodology

Penicillin skin tests were performed by medical laboratory scientists using increasing doses of commercial testing reagents (Diater Laboratories, Madrid. Spain). Skin prick testing (SPT) was followed by intradermal testing (IDT) using a standard protocol with a total of either three or five testing steps, as outlined in the Supplementary Methods. Positive skin testing results were adjudicated by the immunology clinician on the day of the testing based on a wheal size 3 mm greater than a saline control for SPT and an increase of wheal size of 3 mm over the saline control (0.02 ml) for IDT. Antibiotic challenges were performed under observation on the Immunology Day Ward with informed consent and monitoring of vital signs. Challenges were either performed as graded challenges, with the majority of these single-blinded, or as an un-blinded single observed dose. The outcome of the penicillin challenge was adjudicated by an immunology clinician on the day of the challenge. A challenge was determined to be positive when any of the following occurred: conjunctival reaction, respiratory tract symptoms or PEFR/FEV1 decrease ≥15%, cutaneous reactions (pruritus, erythema, urticaria, or angioedema), hypotension, laryngeal oedema, SaO2 fall, or anaphylaxis. A challenge was deemed to be negative if a patient received a dose of 500 mg of amoxicillin, 625 mg (500 + 125 mg) of amoxicillin with clavulanic acid (Augmentin), 500 mg of flucloxacillin, or 500 mg of phenoxymethylpenicillin, and when none of the aforementioned clinical observations occurred.

### Dispensing Data Extract

Electronic community pharmacy antibiotic dispensing records for patients in the study cohort were retrieved from the “TestSafe” pharmacy dispensing record repository; this repository contains community pharmacy dispensing records for patients living in four of the ten District Health Boards in the catchment area for our clinic. Patients can opt to have their dispensing records excluded from the TestSafe database. For these reasons, coverage is not universal in our clinic population. Dispensing records specifically relating to oral and intravenous antibiotics were identified by ATC code (see Supplementary Table 1), grouped into antibiotic classes, and categorized as either occurring prior to or following each individual patient's allergy assessment. Information on topical antibiotics and other systemic antimicrobials, such as antiviral or antifungal medications, was not collected. The pre-assessment time period was defined as the time between the first TestSafe dispensing entry (for any medication, including non-antibiotic medications) and the date of assessment in our clinic (either for an antibiotic challenge or, if skin testing was positive, the date of skin testing). The post-assessment time period was defined as the time between the date of assessment in our clinic and the date of the data extract (19/09/2019).

### Outcomes

The primary outcome of the study was the change in penicillin antibiotic dispensing rates before and after penicillin allergy assessment, stratified by allergy assessment outcome (two groups: penicillin allergy de-labeled and penicillin allergy confirmed). Secondary outcomes, all stratified by allergy assessment outcome, were: the change in non-penicillin antibiotic dispensing rates (including for the non-penicillin antibiotics described in Supplementary Table 1), the change in penicillin antibiotic dispensing measured as a proportion of total antibiotic courses, the change in non-penicillin antibiotic dispensing (including for specific families of penicillin antibiotics outlined in Supplementary Table 1) measured as a proportion of total antibiotic courses, the change in average direct cost of antibiotic, and the change in the proportion of antibiotics that were classified as broad-spectrum.

### Statistical Analyses and Ethical Approvals

Dispensing rates and proportions after assessment were compared with those before assessment using paired *t*-tests and Wilcoxon matched-pairs signed rank tests when data were non-parametric. This study was reviewed and approved by the Auckland Health Research Ethics Committee (AHREC 000130).

## Results

Patients with a positive (evidence of allergy) or negative (no evidence of allergy) penicillin challenge in the Auckland City Hospital Immunology Clinic between 01/01/2012 and 31/12/2017 were identified. There were 483 negative challenges and 17 positive challenges. A review of skin testing records from the same clinic identified 43 patients who were labeled as penicillin allergic based on positive skin test results without an oral challenge. Antibiotic dispensing data, defined as at least one dispensing record for an antibiotic in either the pre- or post- intervention period, was available for 304/483 (62.9%) negative challenge patients, 12/17 (70.6%) positive challenge patients, and 25/43 (58.1%) positive skin testing patients; these 341 patients comprised the study cohort (Table 1), giving 2,955.7 person years of prescribing data that included a total of 6,171 antibiotic dispensings.

**Table 1:** Characteristics of the study groups and their available dispensing data.

	**Penicillin allergy de-labeled**	**Confirmed penicillin allergic**	
	**Negative challenge *n* = 304**	**Positive challenge *n* = 12**	**Positive skin testing *n* = 25**
Age in years (SD)	47.49 (17.94)	43.50 (14.13)	49.52 (17.31)
Female (%)	215 (70.72%)	11 (91.67%)	19 (76%)
Antibiotic used in challenge (%)	Augmentin 280 (92%) Flucloxacillin 9 (2.96%) Amoxicillin 8 (2.63%) Phenoxymethylpenicillin 7 (2.30%)	Augmentin 11 (92%) Flucloxacillin 0 (0%) Amoxicillin 0 (0%) Phenoxymethylpenicillin 1 (8%)	N/A
Person-years of data available pre-assessment	1,255.30	32.66	88.47
Mean duration of dispensing data available pre-assessment (SD)	4.13 years (2.01)	2.72 years (1.04)	3.54 years (1.72)
Total antibiotic courses pre-assessment	2,888	56	289
Person-years of data available post-assessment	1,381.99	68.53	128.76
Mean duration of dispensing data available post-assessment (SD)	4.55 years (1.78)	5.71 years (1.30)	5.15 years (1.51)
Total antibiotic courses post-assessment	2,476	108	354

*Data are shown in three groups: penicillin allergy de-labeled (those who had a negative penicillin challenge) and confirmed penicillin allergic (divided into those with either a positive penicillin challenge or positive penicillin skin testing). There were no significant between group differences in age, duration of dispensing data available pre-assessment, or duration of dispensing data available post-assessment, by one-way ANOVA (with Sidak's multiple comparisons test)*.

**Figure 1:**
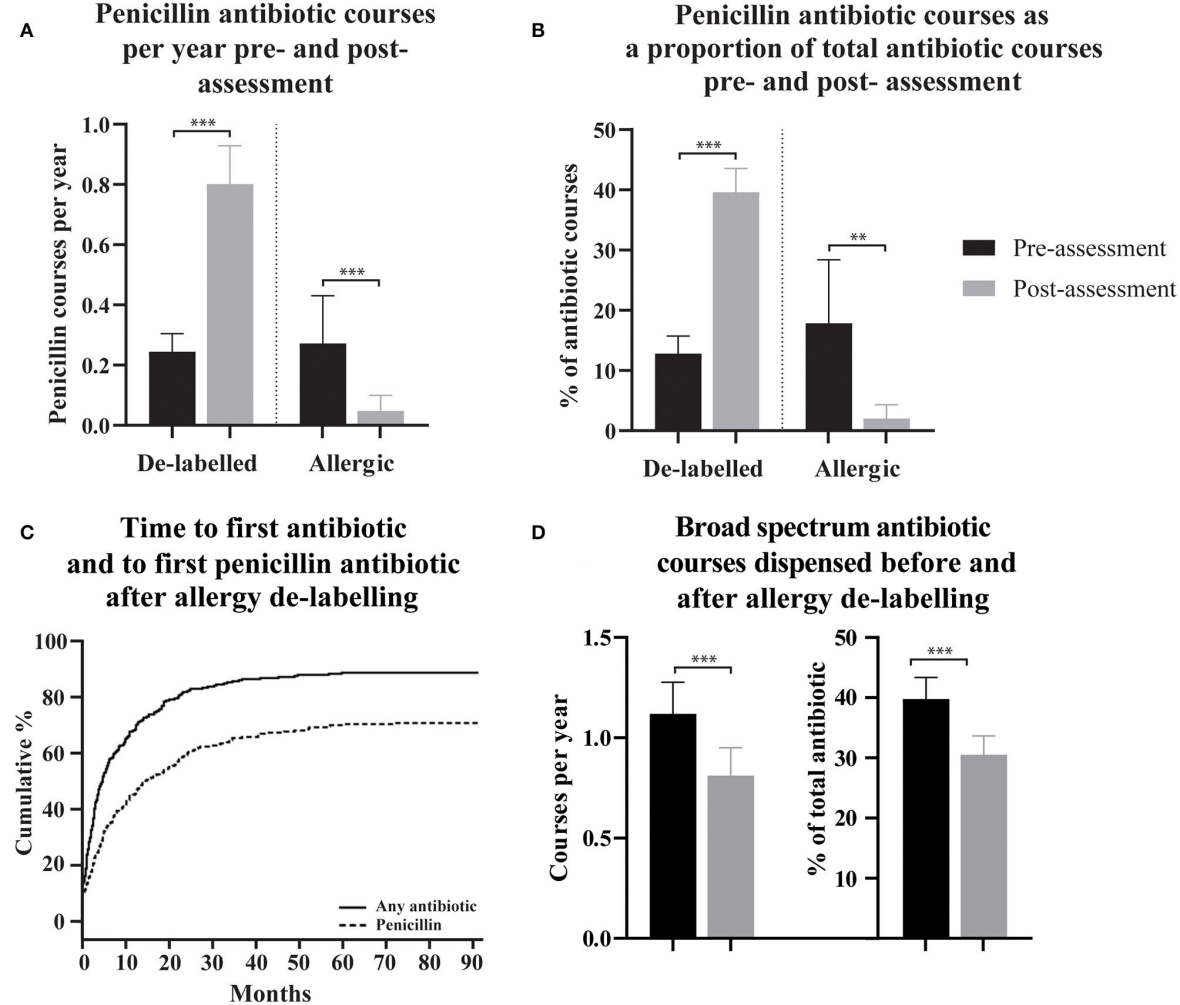
The use of penicillin antibiotics, time to first antibiotic and first penicillin antibiotic, and spectrum of dispensed antibiotic, pre- and post- penicillin allergy de-labeling. **(A,B)** Dispensing rates and proportions of total antibiotic courses are presented as means with 95% confidence intervals for de-labeled patients (*n* = 304) and allergic patients (*n* = 37). **(C)** Cumulative incidence of dispensing for any antibiotic and specifically for a penicillin antibiotic following penicillin allergy de-labeling. **(D)** Broad-spectrum antibiotic dispensing rates and broad-spectrum antibiotic courses as a proportion of each patient's total antibiotics dispensed are presented as a mean with 95% confidence intervals. Dispensing prior to assessment is displayed as black bars **(A,B,D)**. Dispensing following assessment is displayed as gray bars **(A,B,D)**. Antibiotic dispensing rates and specific antibiotic as a proportion of total antibiotics dispensed, before and after de-labeling, were compared using paired *t*-tests (Wilcoxon matched–pairs ranked sign test if data was non-parametric). *P*-values are represented as “ns” for *p* > 0.05, *p ≤ 0.05, **p ≤ 0.01, and ***p ≤ 0.001.

To assess the effect of penicillin allergy assessment, antibiotic dispensing records before and after assessment were compared. In patients who were de-labeled, 215/304 (70.7%) were subsequently dispensed a penicillin antibiotic (Figure 1C), compared with 87/304 (28.6%) in the pre-assessment period; some of these pre-assessment penicillin antibiotic courses are likely to represent the index reaction that resulted in the allergy label. Penicillin antibiotic dispensing rates for the 304 de-labeled patients increased from 0.24 (95% CI 0.18-0.30) penicillin courses per year pre-assessment to 0.80 (95% CI 0.67-0.93) post-assessment (Figure 1A). In contrast, in those with a confirmed penicillin allergy, 5/37 (13.51%) were subsequently dispensed a penicillin antibiotic and dispensing rates in this group decreased from 0.27 (95% CI 0.11–0.43) penicillin courses per year pre-assessment to 0.048 (95% CI 0.00–0.10) post-assessment. There was a reduction in mean total antibiotic courses per year from 2.298 (95% CI 2.06–2.54) to 1.79 (95% CI 1.59–1.99) in de-labeled patients (*n* = 304) and no change in confirmed penicillin allergic patients (*n* = 37), with 2.75 (95% CI 1.59–3.91) and 2.33 (95% CI 1.33–3.33) courses dispensed per year before and after assessment, respectively (Supplementary Figure 1).

For clinical reasons, the total number of annual antibiotic courses is likely to differ between patients. To control for this, dispensing of penicillin antibiotics as a proportion of total antibiotic courses was calculated for each de-labeled patient before and after penicillin allergy de-labeling (*n* = 304, Figure 1B). The mean percentage of a patient's total dispensed antibiotic courses that were penicillin antibiotics increased from 12.81% (95% CI 9.88–15.73) prior to de-labeling to 39.62% (95% CI 35.68–43.57) following de-labeling. In those with confirmed penicillin allergy, the mean percentage of antibiotic courses per patient that were penicillin antibiotics decreased from 17.87% (95% CI 7.34–28.39) to 2.02% (95% CI −0.29–4.32).

**Figure 2:**
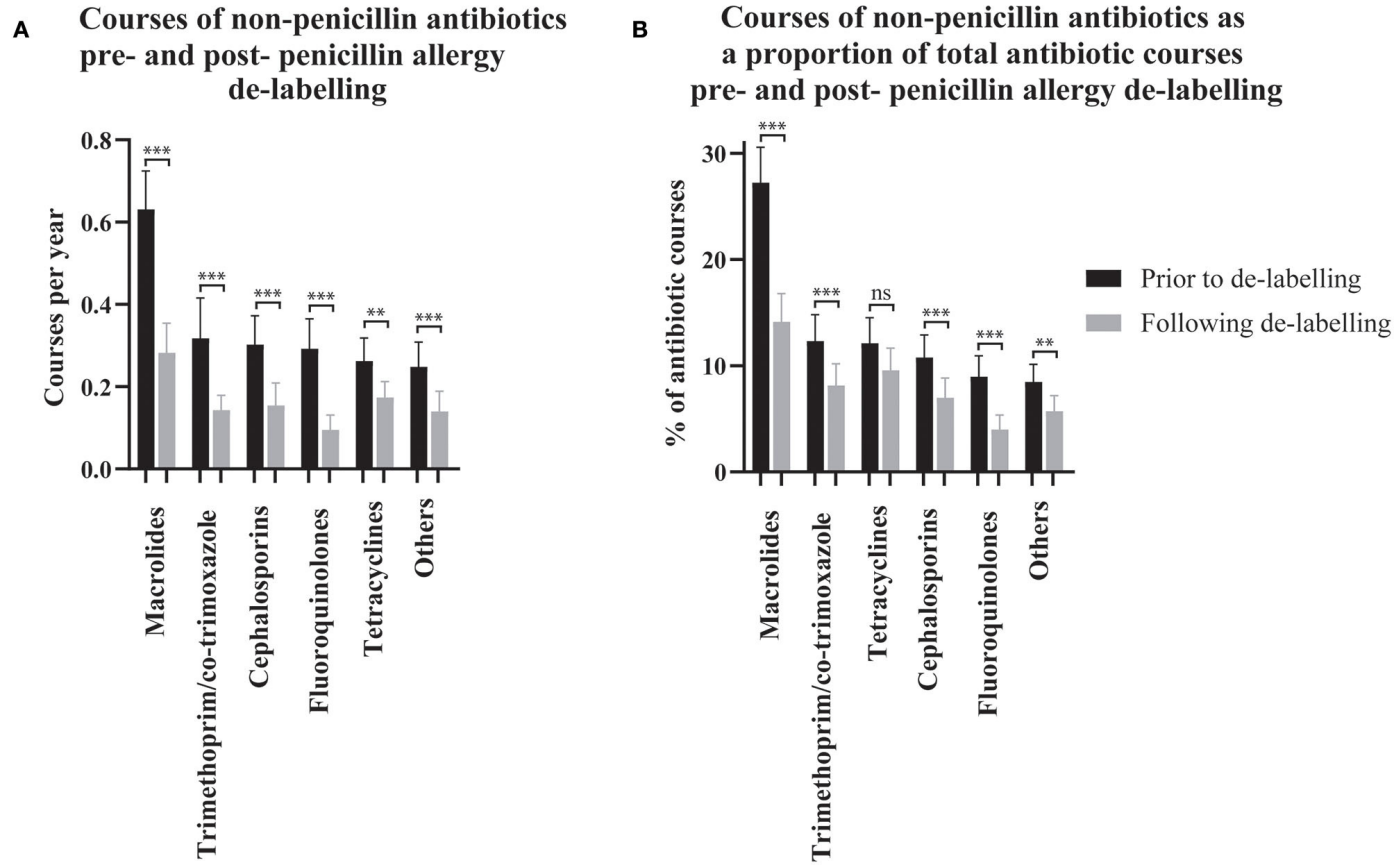
The use of non-penicillin antibiotic pre- and post- penicillin allergy de-labeling. Dispensing rates **(A)** and dispensing as a proportion of each patient's total antibiotic courses **(B)** are presented as means with 95% confidence intervals (*n* = 304 de-labeled patients). Dispensing prior to de-labeling is displayed as black bars. Dispensing following de-labeling is displayed as gray bars. Rates and proportions before and after de-labeling were compared using paired *t*-tests (Wilcoxon matched–pairs ranked sign test if data was non-parametric). P values are represented as “ns” for *p* > 0.05, *p ≤ 0.05, **p ≤ 0.01, and ***p ≤ 0.001.

The effect of penicillin allergy de-labeling on the use of non-penicillin antibiotics was assessed by comparing non-penicillin antibiotic dispensing rates before and after de-labeling (*n* = 304, Figure 2A). Macrolide use reduced from 0.63 (95% CI 0.54–0.72) courses per year pre-assessment to 0.28 (95% CI 0.21–0.35) post-assessment. Taken together, trimethoprim and co-trimoxazole use reduced from 0.32 (95% CI 0.22–0.42) courses per year pre-assessment to 0.14 (95% CI 0.11–0.18) post-assessment. Cephalosporin use reduced from 0.30 (95% CI 0.23–0.37) courses per year pre-assessment to 0.15 (95% CI 0.10–0.21) post-assessment. Fluoroquinolone use reduced from 0.29 (95% CI 0.22–0.36) courses per year pre-assessment to 0.09 (95% CI 0.06–0.13) post-assessment. Tetracycline use reduced from 0.26 (95% CI 0.21–0.32) courses per year pre-assessment to 0.17 (95% CI 0.14–0.21) post-assessment. Other non-penicillin antibiotic use reduced from 0.25 (95% CI 0.19–0.31) courses per year pre-assessment to 0.14 (95% CI 0.09–0.19) post-assessment.

Non-penicillin antibiotic use was also measured as a proportion of each de-labeled patient's total antibiotic use before and after penicillin allergy de-labeling (*n* = 304, Figure 2B). Macrolide use reduced from 27.25% (95% CI 23.94–30.57) to 14.15% (95% CI 11.49–16.81) of dispensed antibiotic courses. Taken together, trimethoprim and co-trimoxazole use reduced from 12.34% (95% CI 9.86–14.81) to 8.14% (95% CI 6.10–10.18) of dispensed antibiotic courses. There was no change in tetracycline use; 12.12% (95% CI 9.70–14.54) vs. 9.56% (95% CI 7.46–11.67) of dispensed antibiotic courses. Cephalosporins use reduced from 10.77% (95% CI 8.65–12.9) to 6.99% (95% CI 5.13–8.84) of dispensed antibiotic courses. Fluoroquinolone use reduced from 8.99% (95% CI 7.04–10.93) to 3.99% (95% CI 2.61–5.36) of dispensed antibiotic courses. Other non-penicillin antibiotic use reduced from 8.49% (95% CI 6.84–10.14) to 5.71% (95% CI 4.23–7.19) of dispensed antibiotic courses. The spectrum of antibiotics dispensed was categorized as described previously (Supplementary Table 1) (23). On average, prior to de-labeling, 39.76% (95% CI 36.14–43.37) of antibiotic courses dispensed to a given patient were broad-spectrum whereas, after de-labeling, 30.53% (95% CI 27.42–33.64) were broad-spectrum (*p* < 0.001, Figure 1D). In a crude analysis, the direct antibiotic cost was $5.78 per course prior to de-labeling and $4.13 per course following de-labeling. The cost of antibiotics was $13.30 per patient-year prior to penicillin allergy de-labeling and $7.38 per patient-year following penicillin allergy de-labeling.

## Discussion

Penicillin allergy de-labeling is increasingly recognized as safe and has the potential to impact antibiotic prescribing. However, longer-term, high-quality data on antibiotic use following penicillin allergy de-labeling is limited. In this pre- and post- study, penicillin antibiotic use increased significantly in those who had their penicillin allergy removed following an observed challenge. After de-labeling, 70.6% of patients went on to use a penicillin antibiotic over a mean follow up period of 4.55 years. Further, patients who had their penicillin allergy confirmed had a lower penicillin antibiotic dispensing rate post-assessment, indicating that there may be benefits of undergoing assessment with skin testing and/or an observed penicillin challenge even for patients who are confirmed to be allergic to penicillin.

Our findings support those from patients enrolled in a study of penicillin skin testing reagents, 94/196 (47%) received at least one penicillin antibiotic in the year following negative skin testing compared with 40/196 (20%) in the year prior (24). At a median follow-up of 15 months following de-labeling by skin testing and oral challenge, 64/163 (39%) de-labeled patients reported that they had taken a beta lactam antibiotic (17). A recent study with longer follow-up (mean 56 months) reported that penicillin antibiotics were taken by 447/639 (70%) de-labeled patients, measured partly by antibiotic purchasing records and partly by self-reported antibiotic use (18).

In de-labeled patients, the increased use of penicillin antibiotics in our study did not result in an overall increase in antibiotic use; total antibiotic use was lower following de-labeling. At 0.80 (95% CI 0.67–0.93) penicillin courses per patient per year, penicillin use in our de-labeled cohort exceeded the national rate of 0.48 penicillin antibiotic courses per year (25), likely reflecting selective referral of patients with high antibiotic requirements to a specialist clinic and the inclusion criteria necessitating an antibiotic dispensing record in the pre- or post- assessment period. As a key limitation, no data on inpatient antibiotic use was available. In our cohort, which is likely to be skewed toward those with a high antibiotic need and frequent contact with the health system, inpatient antibiotic consumption may be higher than the 5% average rate in New Zealand. Penicillin antibiotics are often cheaper than alternatives. In a crude analysis limited to the direct cost of dispensed antibiotics, costs were lower following penicillin allergy de-labeling. This finding is in keeping with previous more detailed health economic research which has shown dramatically reduced health care costs in those who undergo penicillin allergy assessment (26, 27).

This study also demonstrated that non-penicillin antibiotic use, when measured by either dispensing rate or as a proportion of a given patient's total antibiotic courses, reduced significantly across a range of classes that are important targets for antimicrobial stewardship (including cephalosporins, macrolides, fluoroquinolones, and trimethoprim/co-trimoxazole). Further, rates of broad-spectrum antibiotic use were lower following penicillin allergy de-labeling. A reduction in tetracycline use was an inconsistent finding perhaps reflecting that tetracycline has indications outside of the treatment of acute infections (such as for acne) and so may be prescribed specifically in certain situations without consideration given to alternative antibiotics. This represents an important limitation of the study: no data were available on the indication for each antibiotic course as these data are housed in separate health record systems e.g., in primary care, acute clinics, etc. Some antibiotic use captured in our dispensing data will have been for empiric treatment of infections where penicillin antibiotics are first line, others will have been prescribed for infections where other antibiotics are recommended preferentially (e.g., urinary tract infections, where local guideline-based treatment is trimethoprim, or nitrofurantoin) (28), and some will have been directed at microbiology culture and susceptibility results.

The retrospective and observational nature of this study means that other factors could have affected antibiotic use in this cohort and, therefore, changes in antibiotic dispensing patterns cannot solely be attributed to the effect of the penicillin allergy assessment. Results should be interpreted acknowledging that antibiotic dispensing data were used as a surrogate for antibiotic consumption and were available for 341/543 (63%) patients assessed in the Penicillin Allergy Clinic. As a regional service, some patients attending our clinic may reside in areas outside of the greater Auckland region that is covered by the TestSafe dispensing repository. Further, patients can opt to have their dispensing data excluded from the repository and it is possible that some patients did not use any antibiotics in the pre- and post- assessment periods (although referrals to this clinic are usually prompted by a frequent requirement for antibiotics in patients with a penicillin allergy label). Clinical information on the nature of the reaction that gave rise to the penicillin allergy label was not available. We do not know whether subsequent antibiotic courses resulted in allergic or adverse drug reactions. Previous work indicates that in patients with negative skin testing, after a mean follow-up period was 4.5 years, and after exposure to a mean of 8.2 therapeutic antibiotic courses, the rate of new antibiotic “allergy” to penicillin antibiotics was 2.9% (29). Lastly, there was a marked imbalance between the small number of patients determined to be allergic, with a positive challenge or positive skin testing, and the large number of patients who were de-labeled, meaning conclusions regarding antibiotic use in the group with a confirmed penicillin allergy should be interpreted with caution.

In conclusion, this pre- and post- study of the effect of penicillin allergy assessment on antibiotic dispensing patterns demonstrates that penicillin allergy de-labeling is associated with increased penicillin antibiotic use, decreased non-penicillin antibiotic use, and reduced antibiotic costs. This study provides support for penicillin allergy evaluation in a specialist clinic as an important intervention in the era of antimicrobial stewardship. It is not clear if other de-labeling interventions delivered outside a specialist clinic, such as pharmacist-led penicillin allergy de-labeling, which have the potential to improve access to penicillin allergy de-labeling, will be associated with similar changes in antibiotic use although early data are promising (20). Whether the changes in antibiotic use associated with penicillin allergy de-labeling will abrogate other negative clinical outcomes associated with a penicillin allergy label, such as increased rates of infection with multi-resistant organisms, increased length of hospital stay, and increased health care costs, requires further investigation.

## Data Availability Statement

The raw data supporting the conclusions of this article will be made available by the authors, without undue reservation.

## Ethics Statement

The studies involving human participants were reviewed and approved by Auckland Health Research Ethics Committee (AHREC 000130). Written informed consent for participation was not required for this study in accordance with the national legislation and the institutional requirements.

## Author Contributions

TH designed the study, performed data analysis, and wrote the manuscript. NA performed the dispensing data extract and data analysis. ED performed data analysis and wrote the manuscript. JC performed data entry and analysis. AJ designed the study. PF designed the study and wrote the manuscript. All authors contributed to the article and approved the submitted version.

## Conflict of Interest

The authors declare that the research was conducted in the absence of any commercial or financial relationships that could be construed as a potential conflict of interest.

## Abbreviations

PAL: penicillin allergy label
PEFR: peak expiratory flow rate
FEV1: forced expiratory volume in 1 s
SaO_2_: oxygen saturations by pulse oximetry
SPT: skin prick testing
IDT: intradermal testing.
