# Supplementary material for: Penicillin Allergy De-labeling Results in Significant Changes in Outpatient Antibiotic Prescribing Patterns
Source: Front Allergy. 2020 Dec 16;1:586301. doi: 10.3389/falgy.2020.586301 (PMC8974713; doi:10.3389/falgy.2020.586301)
Supplement: Supplementary file 1 [file Data_Sheet_1.PDF]

Supplementary Table 1 – ATC codes and spectrum of antimicrobial activity for oral antibiotics included in this study

**Level 5 ATC code**	**ATC level 5 name**	**Family**	**ECDC Classification** [23]
J01CA04	Amoxicillin	Penicillin	Narrow
J01CR02	Amoxicillin and enzyme inhibitor	Penicillin	Broad
J01CE10	Benzathine phenoxymethylpenicillin	Penicillin	Narrow
J01CF05	Flucloxacillin	Penicillin	Narrow
J01CE02	Phenoxymethylpenicillin	Penicillin	Narrow
J01CA08	Pivmecillinam	Penicillin	Narrow
J01DC04	Cefaclor	Cephalosporin	Broad
J01DB01	Cefalexin	Cephalosporin	Narrow
J01DD13	Cefpodoxime	Cephalosporin	Broad
J01DC02	Cefuroxime	Cephalosporin	Broad
J01MA02	Ciprofloxacin	Fluoroquinolone	Broad
J01MA06	Norfloxacin	Fluoroquinolone	Broad
J01EE01	Sulfamethoxazole and trimethoprim	Trimethoprim and co-trimoxazole	Other
J01EA01	Trimethoprim	Trimethoprim and co-trimoxazole	Other
J01FA10	Azithromycin	Macrolide	Broad
J01FA09	Clarithromycin	Macrolide	Broad
J01FA01	Erythromycin	Macrolide	Narrow
J01FA06	Roxithromycin	Macrolide	Broad
J01AA02	Doxycycline	Tetracycline	Other
J01AA04	Lymecycline	Tetracycline	Other
J01AA07	Tetracycline	Tetracycline	Other
J01FF01	Clindamycin	Other	Broad
J01XD01	Metronidazole	Other	Other
J01XE01	Nitrofurantoin	Other	Other

Supplementary methods

Penicillin skin testing was performed using either a three step or five step protocol with the choice of protocol determined by clinician preference. In the five step protocol, all steps below were performed. In the three step protocol, steps one and three were omitted. Positive skin testing results were adjudicated by the immunology clinician on the day of the testing based on a wheal size 3 mm greater than a saline control for SPT and an increase of wheal size of 3mm over the saline control (0.02 ml) for IDT.

Step 1 Skin Prick Testing Time incubation start: [ ]

	Drug	Conc.	Unit	Wheal (mm)	Flare (mm)
1	Negative Control (Saline)	0.9	%		
2	Histamine Control	10	mg/mL		
3	Benzyl Penicillin	0.6	mg/mL		
4	Major Determinant (PPL)	0.004	mg/mL		
5	Minor Determinant (MD)	0.05	mg/mL		
6	Amoxycillin	2	mg/mL		
7	Amoxycillin / Clavulanic acid	2	mg/mL		
8	Flucloxacillin	1	mg/mL		

Step 2 Skin Prick Testing Time incubation start: [ ]

9	Benzyl Penicillin	6	mg/mL		
10	Major Determinant (PPL)	0.04	mg/mL		
11	Minor Determinant (MD)	0.5	mg/mL		
12	Amoxycillin	20	mg/mL		
13	Amoxycillin / Clavulanic acid	20	mg/mL		
14	Flucloxacillin	10	mg/mL		

Step 3 Intradermal Testing Time incubation start: [ ]

15	Negative Control (Saline)	0.9	%		
16	Benzyl Penicillin	0.06	mg/mL		
17	Major Determinant (PPL)	0.0004	mg/mL		
18	Minor Determinant (MD)	0.005	mg/mL		
19	Amoxycillin	0.2	mg/mL		
20	Amoxycillin / Clavulanic acid	0.2	mg/mL		
21	Flucloxacillin	0.1	mg/mL		

Step 4 Intradermal Testing Time incubation start: [ ]

22	Benzyl Penicillin	0.6	mg/mL		
23	Major Determinant (PPL)	0.004	mg/mL		
24	Minor Determinant (MD)	0.05	mg/mL		
25	Amoxycillin	2	mg/mL		
26	Amoxycillin / Clavulanic acid	2	mg/mL		
27	Flucloxacillin	1	mg/mL		

Step 5 Intradermal Testing Time incubation start: [ ]

28	Benzyl Penicillin	6	mg/mL		
29	Major Determinant (PPL)	0.04	mg/mL		
30	Minor Determinant (MD)	0.5	mg/mL		
31	Amoxycillin	20	mg/mL		
32	Amoxycillin / Clavulanic acid	20	mg/mL		
33	Flucloxacillin	10	mg/mL		
