# Supplementary figures and images for: Penicillin Allergy De-labeling Results in Significant Changes in Outpatient Antibiotic Prescribing Patterns
Source: Front Allergy. 2020 Dec 16;1:586301. doi: 10.3389/falgy.2020.586301 (PMC8974713; doi:10.3389/falgy.2020.586301)

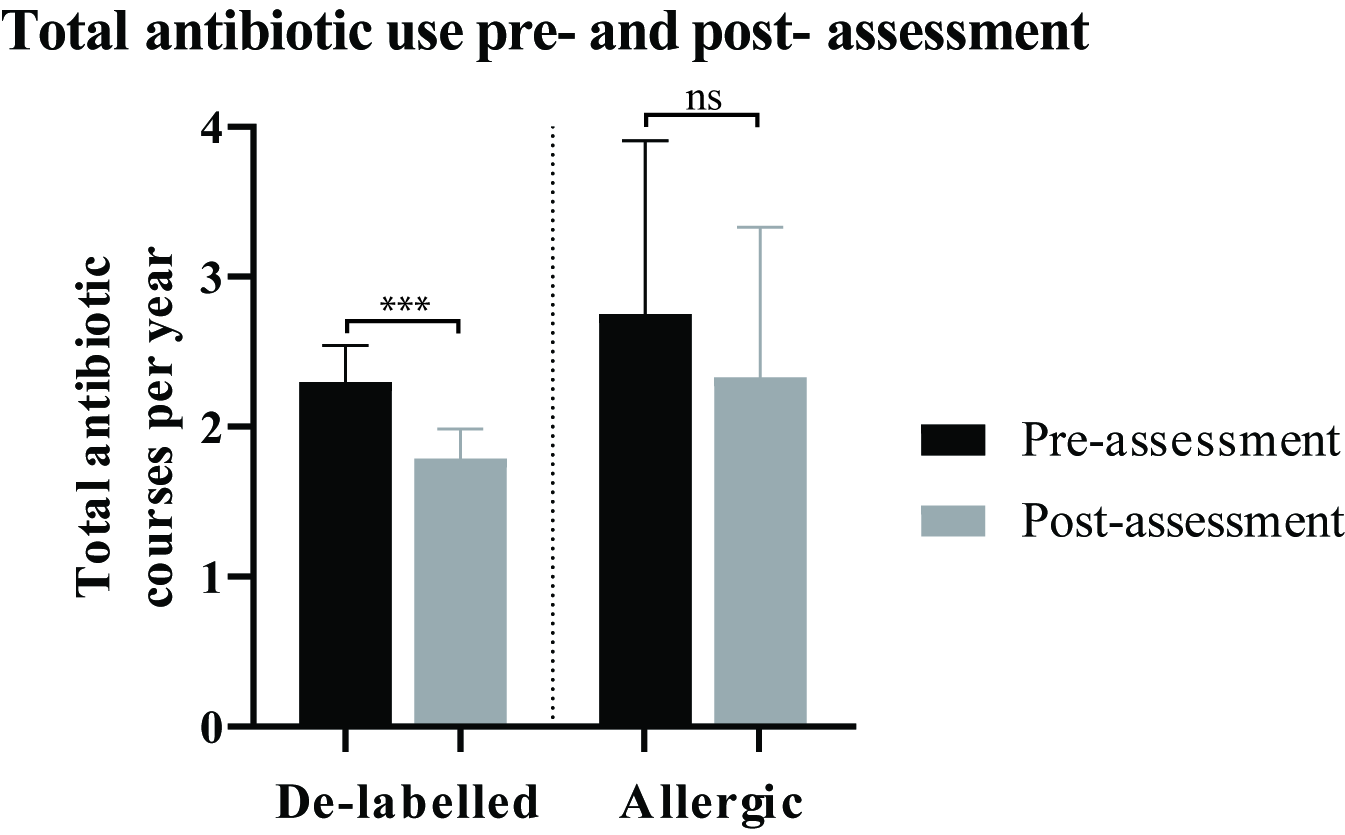

Supplement: Supplementary file 2 [file Image_1.TIF]
